# Vascular Endothelial Cell Injury Is an Important Factor in the Development of Encapsulating Peritoneal Sclerosis in Long-Term Peritoneal Dialysis Patients

**DOI:** 10.1371/journal.pone.0154644

**Published:** 2016-04-27

**Authors:** Mitsuhiro Tawada, Yasuhiko Ito, Chieko Hamada, Kazuho Honda, Masashi Mizuno, Yasuhiro Suzuki, Fumiko Sakata, Takeshi Terabayashi, Yoshihisa Matsukawa, Shoichi Maruyama, Enyu Imai, Seiichi Matsuo, Yoshifumi Takei

**Affiliations:** 1 Department of Nephrology and Renal Replacement Therapy, Nagoya University Graduate School of Medicine, Nagoya, Japan; 2 Department of Nephrology, Juntendo University, Tokyo, Japan; 3 Department of Pathology, Tokyo Women’s Medical University, Tokyo, Japan; 4 Department of Urology, Nagoya University Graduate School of Medicine, Nagoya, Japan; 5 Department of Biochemistry, Nagoya University Graduate School of Medicine, Nagoya, Japan; Robert Bosch Hospital, GERMANY

## Abstract

**Background and Objectives:**

Encapsulating peritoneal sclerosis (EPS) is a rare but serious and life-threatening complication of peritoneal dialysis (PD). However, the precise pathogenesis remains unclear; in addition, predictors and early diagnostic biomarkers for EPS have not yet to be established.

**Methods:**

Eighty-three peritoneal membrane samples taken at catheter removal were examined to identify pathological characteristics of chronic peritoneal deterioration, which promotes EPS in patients undergoing long-term PD treatment with low occurrence of peritonitis.

**Results:**

According to univariable logistic regression analysis of the pathological findings, thickness of the peritoneal membrane (*P* = 0.045), new membrane formation score (*P* = 0.006), ratio of luminal diameter to vessel diameter (L/V ratio, *P*<0.001), presence of CD31-negative vessels (*P* = 0.021), fibrin deposition (*P*<0.001), and collagen volume fraction (*P* = 0.018) were associated with EPS development. In analyses of samples with and without EPS matched for PD treatment period, non-diabetes, and PD solution, univariable analysis identified L/V ratio (per 0.1 increase: odds ratio (OR) 0.44, *P* = 0.003) and fibrin deposition (OR 6.35, *P* = 0.027) as the factors associated with EPS. L/V ratio was lower in patients with fibrin exudation than in patients without fibrin exudation.

**Conclusions:**

These findings suggest that damage to vascular endothelial cells, as represented by low L/V ratio, could be a predictive finding for the development of EPS, particularly in long-term PD patients unaffected by peritonitis.

## Introduction

Encapsulating peritoneal sclerosis (EPS) is a rare but life-threatening complication of peritoneal dialysis (PD), and the precise pathogenesis remains obscure [1–3]. Most cases of EPS develop after the termination of PD [4] and are associated with a longer duration of PD treatment [2,4–6], peritonitis [1,6,7], and high peritoneal transport with rapid disappearance of osmotic conductance [2,7–10]. In addition, high cumulative glucose exposure and levels of glucose degradation products [9], young age [7,10], and kidney transplantation [7,11,12] have been reported as risk factors. Abdominal computed tomography (CT) is an established diagnostic tool for EPS, but is not useful to predict the development of EPS as a subclinical condition [13,14]. Biomarkers such as decreased levels of cancer antigen 125 and increased levels of interleukin-6 in peritoneal effluent, either singly or in combination, could be useful in some cases [15]. C-reactive protein levels are also reportedly increased within the year before EPS diagnosis [16]. Although several studies have been reported to date, including the above-mentioned paper [15], neither predictors nor early diagnostic measures of EPS have yet been established [1,2,17]. Pathological findings for EPS have recently been studied in detail [18,19], but no published studies have explored pathological findings predictive of EPS development using peritoneal membrane specimens taken at the time of PD discontinuation.

The present study sought to identify predictive findings of EPS by analyzing pathological findings in peritoneal membrane tissues taken at catheter removal. In particular, we focused on pathological findings indicative of chronic peritoneal deterioration, which would promote EPS in long-term PD patients unaffected by peritonitis. This is the first report to investigate predictors for EPS using peritoneal biopsy tissues obtained at the cessation of PD.

## Materials and Methods

### Patient profiles and demographic data

This study was approved by the Ethics Committee for Human Research of the Faculty of Medicine at Nagoya University (Approval number 299) and Juntendo University (Approval number 26–010). Informed consent was obtained from all patients. A flow diagram of the study population is shown in S1 Fig.

A total of 368 peritoneal biopsy specimens were screened, taken from the Department of Nephrology and Renal Replacement Therapy at Nagoya University Hospital (Nagoya, Japan), hospitals affiliated with Nagoya University, and Juntendo University Hospital (Tokyo, Japan). All patients were Japanese and over 18 years of age. In order to evaluate the pathological findings at the time of removal of the PD catheter as a predictor of EPS, 178 biopsy samples from pre-dialysis patients were excluded from this study. Tissue samples obtained at the time of catheter removal for reasons of peritonitis or if the patient had experienced an episode of peritonitis within the past 1 month were also excluded (n = 46). In addition, 61 peritoneal biopsy samples were judged as not appropriate for this study because conditions of the samples were not suitable in assessment of size, site, direction, or damage of the specimens, including lack of peritoneal surface membrane, according to the paper by Honda et al. [20]. Data on the underlying causes of end-stage renal disease, demographic details, duration of PD treatment, occurrence of peritonitis, prescription of steroids, use of acidic glucose-based PD solution (Baxter, Tokyo, Japan), acidic icodextrin PD solution (Baxter), and peritoneal lavage before removal of PD catheter [21] were collected from the medical records. Causes of renal failure were chronic glomerulonephritis (n = 34, 41.0%), diabetic nephropathy (n = 22, 26.5%), nephrosclerosis (n = 9, 10.8%) and others (n = 18, 21.7%). The glucose exposure score was defined by the sum of the scores of all PD solutions used in a day. Scores were defined as follows: Score 0, 1.5% PD solution; Score 1, 2.5% PD solution; and Score 2, 4.25% PD solution.

### Definition of EPS

Diagnosis of EPS in this study was performed based on the clinical features of gastrointestinal obstruction due to bowel obstruction and features of encapsulation with peritoneal fibrosis, according to the position paper on EPS by the International Society for Peritoneal Dialysis [2].

### Processing of biopsy samples and immunohistochemistry

Parietal peritoneal tissues were biopsied in the standard manner and processed as reported previously [22,23]. Parietal peritoneal membrane was obtained from the anterior abdominal wall when the PD catheter was removed at the cessation of PD. To avoid detachment of mesothelial cells, all procedures were carried out carefully. Peritoneal membrane tissues fixed with formalin were stained with hematoxylin and eosin (HE) and Masson’s trichrome [23–26] and were also stained with phosphotungstic acid hematoxylin (PTAH) reagent to detect fibrin formation, as described previously [27]. Immunostaining was performed on paraffin-embedded tissues as described previously [23–25,28]. The antibodies used in these experiments are summarized in S1 Table. Briefly, 4-μm-thick sections of formalin-fixed, paraffin-embedded tissues were dewaxed and rehydrated. Endogenous peroxidase activity was inhibited with 3% H_2_O_2_ in methanol. For antigen retrieval to detect CD31 and CD68, the slides were boiled in a solution of 0.04 M citrate and 0.12 M phosphate (pH 5.8) for 30 min at 98°C. After washing, nonspecific protein-binding sites were blocked with normal goat serum (Dako, Glostrup, Denmark). Then, sections were incubated with primary antibodies, mouse monoclonal antibodies against CD31 (JC/70A; Dako), CD68 (PGM1; Dako), and podoplanin (D2-40; Dako) overnight at 4°C. For advanced glycation end-products (AGEs), sections were incubated with mouse anti-AGE antibody (6D12; TransGenic, Kobe, Japan) for 60 min at room temperature. After washing with phosphate-buffered saline, sections were treated with a conjugate of polyclonal goat anti-mouse immunoglobulin (Ig) G antibodies and horseradish peroxidase-labeled polymer (Histofine^®^ Simple Stain; Nichirei, Tokyo, Japan) as the secondary reagent. Enzyme activity was detected by 3,3'-diaminobenzidine (Nichirei). For analysis of collagen volume fraction (collagen density), we applied the methods described by Morelle et al. [29]. Briefly, we stained peritoneal membrane tissues with a Picrosirius Red Stain kit (Polyscience, Warrington, PA), then observed the tissues under circularly polarized light microscopy (Zeiss Z1 image microscopy, Carl Zeiss, Oberkochen, Germany). Collagen volume fraction was assessed using ImageJ software version 1.5 (http://imagej.nih.gov/ij/) and was calculated as a percentage of the submesothelial area.

### Morphological analysis

The 144 peritoneal biopsy samples taken at cessation of PD and catheter removal included those from 13 patients who developed EPS after catheter removal (S1 Fig). The adequacy of samples was judged as described in the report by Honda et al. [20], in which <50% of samples were considered to be appropriate. Sixty-one samples, including 3 cases with occurrence of EPS, were unsuitable for assessing pathological findings in the present study. In order to assess the extent of peritoneal thickening, the submesothelial compact zone was defined and thickness was measured at 5 points; the mean was calculated as described previously [20] (S2A Fig). For evaluation of CD31-positive vessels and CD68-positive macrophages, all tissue samples were scanned using a ScanScope^®^ AT Turbo scanner (Leica Biosystems, Nussloch, Germany) and examined in the submesothelial compact zone using ImageScope software (Leica Biosystems). Densities of inflammatory cells and blood vessels were calculated and expressed as the number per peritoneal surface length (per millimeter), as described previously [22,23]. Vasculopathy was assessed by severity of luminal narrowing and by the ratio of luminal diameter to vessel diameter (L/V ratio) at the level of the postcapillary venules, as described previously [20,30] (S2B Fig). Mean L/V ratio in the postcapillary venules was defined as the “L/V ratio” in the present study. “New membrane formation” is defined as an extra-thin fibrinous membrane that encapsulates the outer surface of the original peritoneal membrane [31] and is identical to the capsular membrane [19]. “New membrane formation” was reported in both EPS and non-EPS fibrotic peritoneal membrane [31]. “New membrane formation” was assessed by a positive percentage of surface length and thickness. Positive percentage of surface length was graded into 4 groups: 0, 0%; 1, >0% and ≤25%; 2, >25% and ≤50%; or 3, >50% and ≤100%. Thickness of the new membrane was likewise graded into 4 groups: 0, 0; 1, >0 and ≤100 μm; 2, >100 and ≤250 μm; or 3, >250 μm. The mean of these two grades was defined as the new membrane formation score (S2C Fig). Podoplanin (D2-40)-positive cells were semi-quantitatively classified into three groups according to the reports by Braun [18]: 0) positive podoplanin staining on lymphatics and mesothelial cells, but not on single cells with fibroblastic appearance; 1) focal accumulation of podoplanin-positive cells with fibroblastic appearance; and 2) diffuse accumulation of podoplanin-positive cells with fibroblastic appearance (S2D Fig). AGE accumulation [32]was analyzed in the interstitial area and in the vessel walls separately and semi-quantitatively classified into 4 grades based on the intensity of positive staining: 0, no staining; 1, mild staining; 2, moderate staining; and 3, pronounced staining. Mean scores were then calculated and defined as the AGE accumulation score (S2E Fig). The presence rate of mesothelial cells was assessed as a positive percentage for total surface length and was graded into 4 groups: 0, 0%; 1, >0% and ≤25%; 2, >25% and ≤50%; and 3, >50% and ≤100% (S3A and S3B Fig). Perivascular bleeding (S3C and S3D Fig), positivity of fibrin deposition detected by PTAH staining [27] (S3E and S3F Fig), and presence of negative staining for CD31 in blood vessels (S3G and S3H Fig) were assessed as presence (+) or absence (-).

### Statistical analysis

The Shapiro-Wilk test was applied to test normal distributions. Variables with a normal distribution are expressed as mean values ± standard deviation (SD), and asymmetrically distributed data are given as median and interquartile range. Categorical variables are presented as numbers and percentages. To examine differences between two independent groups, Student’s *t*-test (for normally distributed variables) and the Mann-Whitney U-test (for non-normally distributed variables) were used. Fisher’s exact test was employed when variables were categorical. In consideration of the quite small sample size in the present study, we analyzed the data by Firth’s logistic regression analysis [33] to identify predictors of EPS. Using the variables showing significance on univariable analysis, additional analyses were undertaken to explore independent predictors of EPS using Firth’s multivariable logistic regression to obtain odds ratios with 95% confidence intervals (CIs). To address multicollinearity among independent variables of the multivariable model, Spearman’s rank correlation coefficient was calculated, and each highly correlated independent variable was evaluated separately in different models. Variables were selected by the backwards stepwise method, and the best model was chosen according to the Akaike information criterion. The candidates for predictors of EPS that were assessed were as follows: age, PD duration, diabetic nephropathy, acidic PD solution, peritoneal lavage, steroid treatment, glucose exposure score, use of icodextrin, and number of episodes of peritonitis as clinical predictors; thickness of peritoneal membrane, CD68-positive cell count, new membrane formation, D2-40 expression, presence of mesothelial cells, perivascular bleeding, L/V ratio, expression of CD31-positive vessels, presence of CD31-negative vessels, fibrin deposition, AGE score, and collagen volume fraction as pathological predictors. In addition, we compared general characteristics and pathological findings between the EPS and non-EPS development groups matched for PD treatment period, diabetes, and PD solution (acidic or neutral pH) by conditional logistic regression with Firth’s bias correction. Statistical analysis was performed with IBM SPSS Statistics version 22.0 (International Business Machines Corporation, Armonk, NY) and R version 3.0.2 (R Core Team (2013). R: A language and environment for statistical computing. R Foundation for Statistical Computing, Vienna, Austria. URL http://www.R-project.org/). Values of *P*<0.05 were considered significant.

## Results

### General characteristics of the study cohort

**Fig 1:**
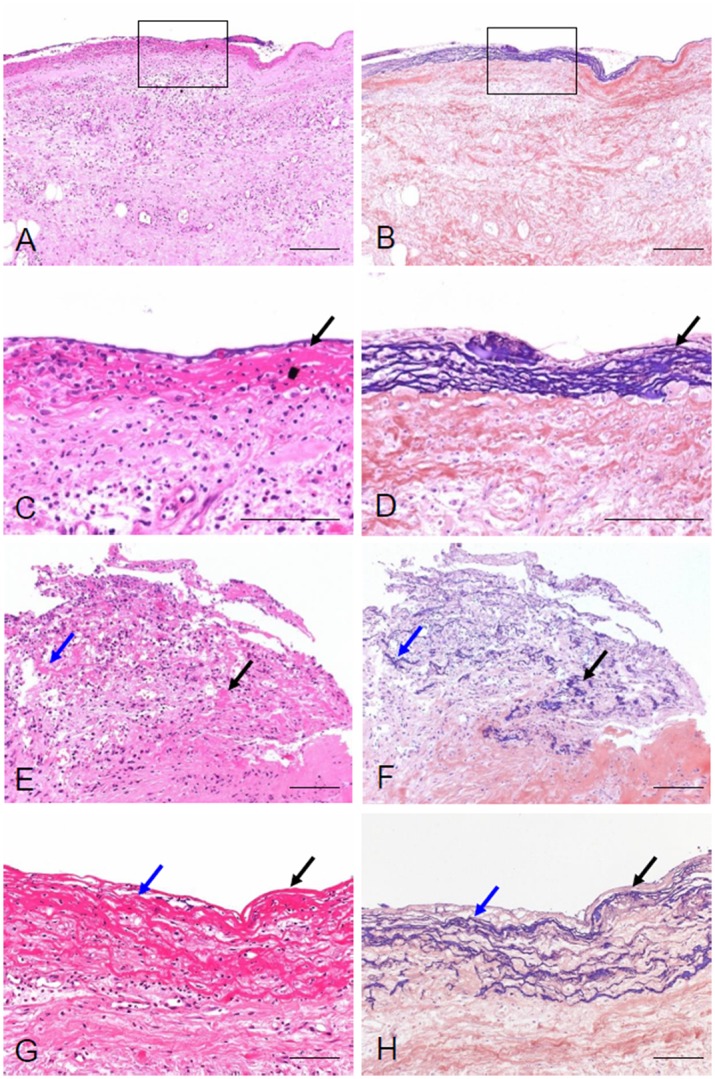
Representative pathological findings of severe peritonitis, which is associated with fibrin exudation. Fungal peritonitis (**A**-**D**), *Pseudomonas aeruginosa* peritonitis (**E**, **F**), and *Serratia marcescens* peritonitis (**G, H**) show exudation of fibrin on the surface of peritoneal membrane associated with inflammatory cell infiltration. **B, D**, **F, and H** are serial sections of panels **A, C, E,** and **G**, respectively. **C** and **D** show higher-magnification images of the boxed areas in **A** and **B**. Black and blue arrows indicate the same areas in serial sections. **A**, **C**, **E**, and **G:** HE staining; **B**, **D**, **F,** and **H:** PTAH staining. Scale bars in **A** and **B** = 200 μm. Scale bars in **C** to **H** = 100 μm.

**Fig 2:**
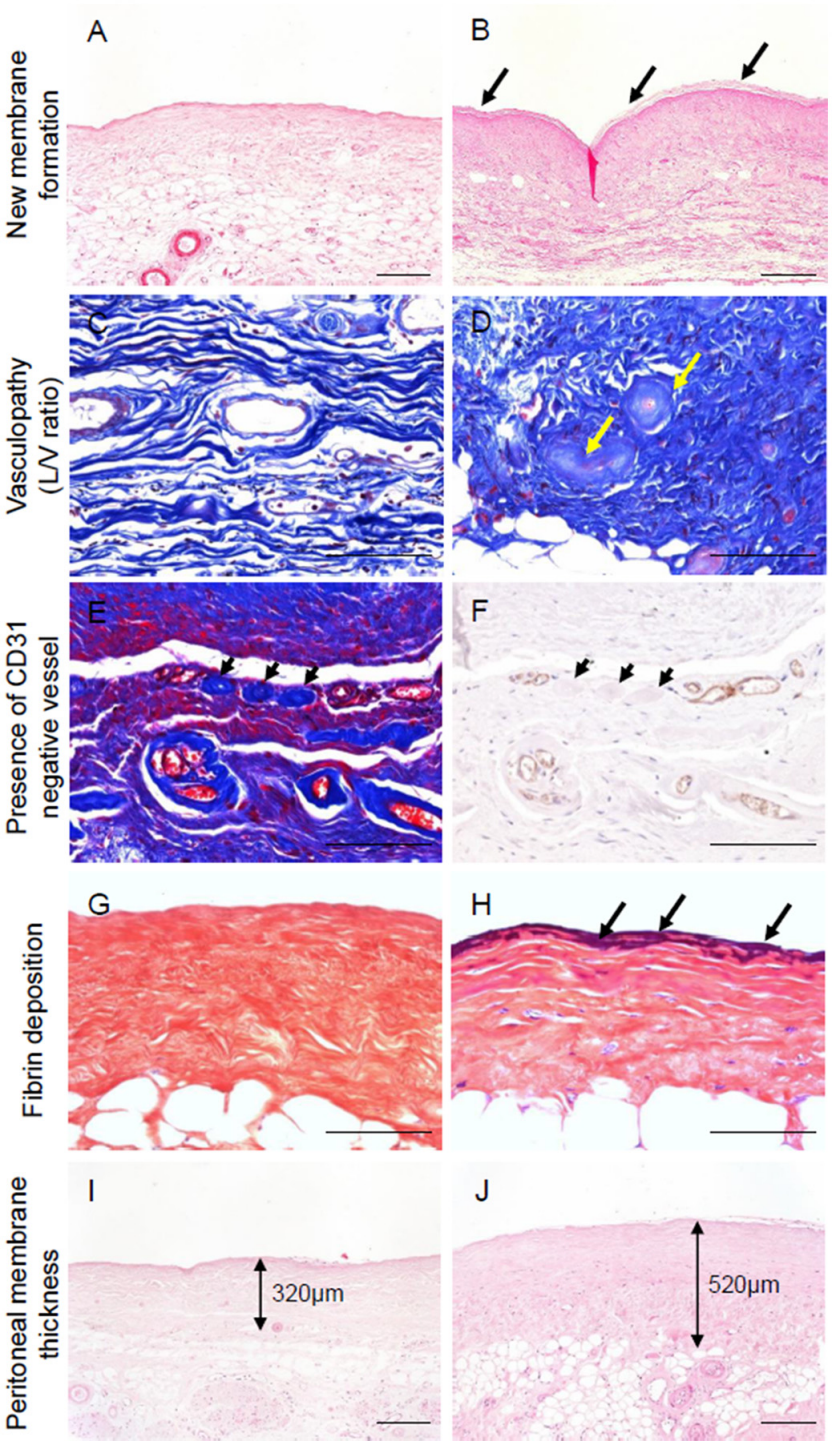
Pathological findings of peritoneal membrane taken at PD catheter removal for predicting EPS. **A, B: Presence of new membrane formation.** Negative (**A**), positive (**B**). Arrows indicate new membrane formation. **C, D: Vasculopathy assessed by luminal diameter to vessel diameter (L/V) ratio at the level of the postcapillary venules.** (**C**) Mild vasculopathy (L/V ratio = 0.78). (**D**) Severe vasculopathy (arrows, L/V ratio = 0–0.14). **E, F: Presence of CD31-negative vessels.** Arrows indicate negative staining for CD31 (**F**) due to severe endothelial cell damage. **G, H: Fibrin deposition.** Fibrin deposition (arrows) was demonstrated by PTAH staining (**H**). **I, J: Peritoneal thickness.** Thickened peritoneum is prone to be high occurrence of EPS (**J**). **A, B, I,** and **J, HE staining; C, D** and **E,** Masson’s trichrome staining; **F,** CD31 immunostaining; **G, H,** PTAH staining. Scale bars in A, B, I, and J = 200 μm. Scale bars in C to H = 100 μm.

**Fig 3:**
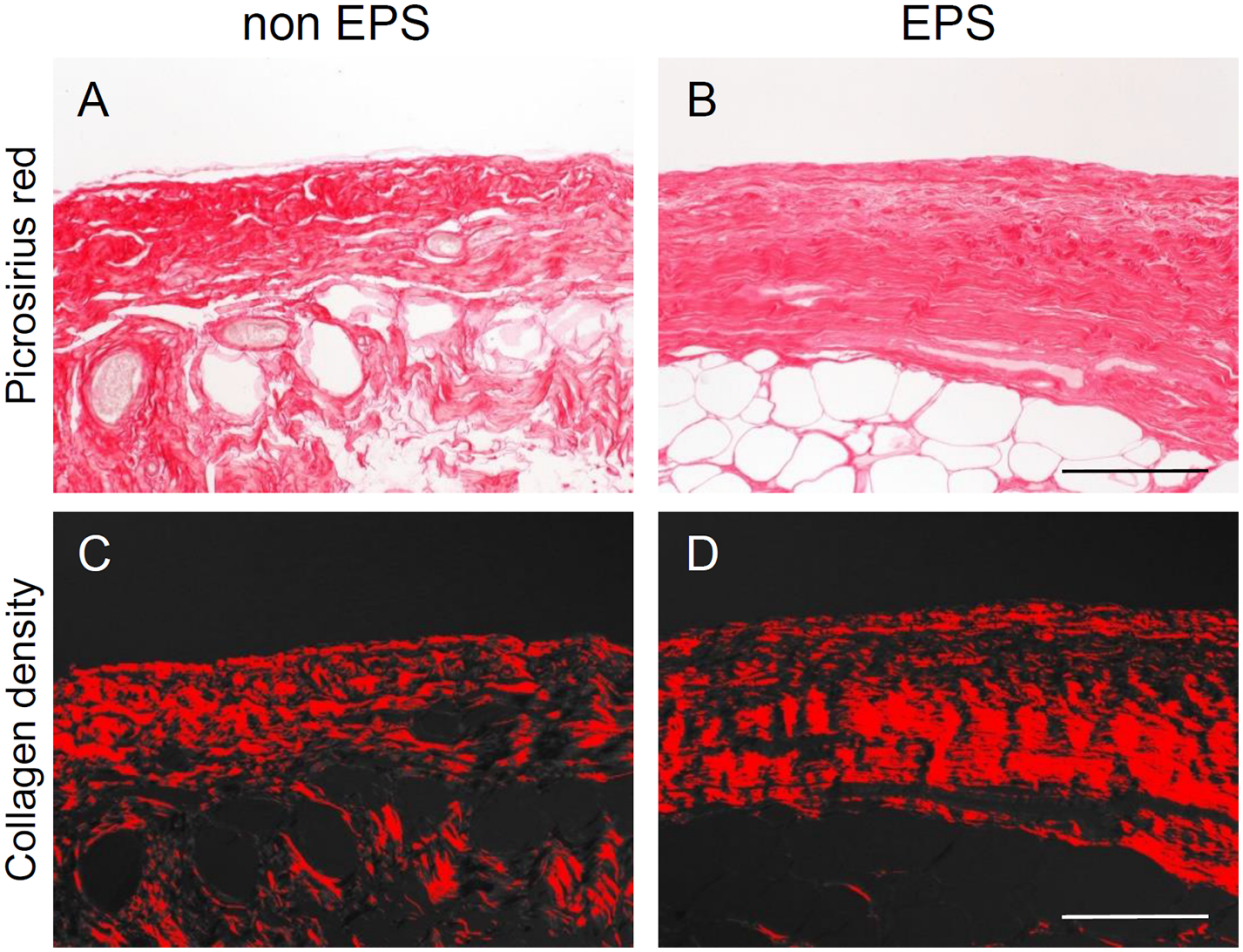
Collagen volume fraction (collagen density) in submesothelial compact zone is higher in the EPS development group than in non-EPS development group. **A, B:** Picrosirius red stain, **C, D:** Collagen volume fraction (collagen density). **A, C:** non-EPS development group, **B, D:** EPS development group. **Scale bars = 200 μm.**

A total of 368 peritoneal membrane biopsy samples were screened (S1 Fig). After excluding pre-dialysis tissue samples, we excluded biopsy samples of peritoneal membranes taken at the cessation of PD because of peritonitis. Nineteen of the 46 peritonitis cases, including seven with difficulty of access because of insufficient tissue size or lack of peritoneal surface, showed fibrin exudation on the surface of the peritoneum (Fig 1). These changes can induce adhesions in the peritoneal cavity, as reported in human and animal experiments [34–36]; we therefore excluded cases with peritonitis. Eighty-three biopsy samples were assessed to identify potential predictors of EPS. We followed-up subsequent EPS occurrence for at least 2 years after the cessation of PD. The details of the clinical factors and pathological findings in patients with and without EPS are shown in Table 1. No patients had symptoms of EPS at catheter removal. Clinical characteristics of the patients who developed EPS were younger age (*P* = 0.006), chronic glomerulonephritis as the primary cause of renal disease (*P* = 0.016), acidic PD solution (*P* = 0.011), longer duration of PD treatment (*P* = 0.003), and high glucose exposure (*P* = 0.002). Pathological characteristics of the patients who developed EPS were presence of new membrane formation (*P* = 0.001, Fig 2A and 2B), low L/V ratio (*P*<0.001, Fig 2C and 2D), presence of CD31-negative vessels (*P* = 0.027, Fig 2E and 2F), fibrin deposition (*P*<0.001, Fig 2G and 2H), and collagen volume fraction (*P* = 0.03, Fig 3). No significant differences between groups were seen in thickness of the peritoneal membrane, presence of mesothelial cells, expressions of podoplanin or CD68. The interval between catheter removal and development of EPS was 9.6±8.7 months.

**Table 1:** General characteristics and pathological findings.

	non-EPS (n = 73)	EPS (n = 10)	*P*-value
Clinical factors			
Age (years)	53.1±12.39	41.2±14.34	0.006 †
Male, n (%)	50 (68.5)	8 (80.0)	0.716 *
Primary kidney disease			
Chronic glomerulonephritis, n (%)	26 (35.6)	8 (80.0)	0.016 *
Diabetes nephropathy, n (%)	22 (30.1)	0 (0.0)	
PD solution			
Neutral solution, n (%)	31 (42.5)	0 (0.0)	0.011 *
Acidic solution, n (%)	42 (57.5)	10 (100)	
PD duration (month)	67 (37.0–100.0)	128 (89.0–149.0)	0.003 ‡
Number of peritonitis (per patient)	0 (0.0–1.0)	1 (0.0–4.0)	0.152 ‡
Peritonitis rate (patient^-1^ × year^-1^)	0.0 (0.00–0.23)	0.1 (0.00–0.25)	0.465 ‡
Use of icodextrin, n (%)	32 (43.8)	7 (70.0)	0.178 *
Glucose exposure score at cessation of PD	1.0 (0.0–2.0)	3.0 (2.0–4.0)	0.002 ‡
Peritoneal lavage, n (%) #1.	41 (56.2)	7 (70.0)	0.507 *
Steroid treatment, n (%) #2.	5 (6.8)	3 (30.0)	0.052 *
Reason for withdrawal from PD			
Dialysis failure/UF failure, n (%)	38 (52.1)	7 (70.0)	0.574 *
Prevention of EPS, n (%) #3.	25 (34.2)	3 (30.0)	
CRP at cessation of PD (mg/dl)	0.19 (0.070–0.400)	0.18 (0.030–0.600)	0.773 ‡
Pathological factors			
Thickness of peritoneal membrane (μm)	330.1 (239.19–430.00)	550.5 (227.77–750.00)	0.114 ‡
CD68 positive cells (number/mm)	13.4 (5.02–21.76)	8.6 (0.56–15.42)	0.226 ‡
Presence of mesothelial cells (grade)	1.0 (0.0–3.0)	1.0 (0.0–3.0)	0.843 ‡
New membrane formation (grade)	0.0 (0.0–0.0)	1.5 (0.0–2.5)	0.001 ‡
D2-40 expression (grade)	0.0 (0.0–0.0)	0.5 (0.0–1.5)	0.050 ‡
CD31 positive vessels (number/mm)	13.9 (10.00–17.31)	11.4 (5.56–14.75)	0.109 ‡
CD31 negative vessels, positive case, n (%)	12 (16.4)	5 (50.0)	0.027 *
Ratio of luminal diameter to vessel diameter (L/V ratio)	0.7 (0.54–0.74)	0.3 (0.26–0.43)	<0.001 ‡
Fibrin deposition, positive case, n (%)	2 (2.7)	5 (50.0)	<0.001 *
Perivascular bleeding, positive case, n (%)	8 (11.0)	2 (20.0)	0.345 *
AGEs score (grade)	2.0 (1.5–2.5)	2.8 (1.5–3.0)	0.071 ‡
Collagen volume fraction (%)	20.4 (13.90–29.55)	30.9 (25.50–48.00)	0.030 ‡

EPS, encapsulating peritoneal sclerosis; PD, peritoneal dialysis; AGEs, advanced glycation end-products; D2-40 is same as podoplanin.

*n (%), Fisher's exact test;

^†^ mean±SD, Student’s t-test;

^‡^ median (IQR), Mann-Whitney's U-test

^#1^. Duration of peritoneal lavage was longer in the EPS-development group (25.0 months (7.0–46.5), n = 41) than in the non-EPS-development group (7.5 months (3.5–15.0), n = 7).

^#2^. In all patients, the initial dose of corticosteroid was 10–20 mg/day, started after stopping PD.

^#3^. “Prevention of EPS” indicates withdrawal from PD based on the recommendation, because long-term PD is a risk factor for development of EPS [3].

### Clinical factors predictive of EPS

Univariable analysis (Table 2A) identified clinical factors that were associated with EPS, including age (OR 0.93, *P* = 0.007), PD duration (OR 1.02, *P* = 0.003), diabetic nephropathy (OR 0.11, *P* = 0.039), acidic PD solution (OR 15.57, *P* = 0.006), steroid treatment (OR 5.81, *P* = 0.034), and glucose exposure score (OR 2.05, *P* = 0.002). Firth’s multivariable logistic regression [33] suggested that a high glucose exposure score was independently associated with EPS (OR 2.03 [95%CI 1.17–3.96], *P* = 0.011) (S2 Table).

**Table 2:** Logistic regression analysis of clinical and pathological predictors for EPS.

**A. Clinical predictors for EPS**		
	Univariable Logistic Regression	
Factors	OR (95%CI)	*P*
Age (per 1-year increase)	0.93 (0.87–0.98)	0.007
PD duration (per 1-month increase)	1.02 (1.01–1.04)	0.003
Diabetes nephropathy	0.11 (0.00–0.91)	0.039
Acidic PD solution	15.57 (1.88–2029.29)	0.006
Peritoneal lavage	1.68 (0.46–7.37)	0.443
Steroid treatment	5.81 (1.16–27.14)	0.034
Glucose exposure score at cessation of PD (per 1-score increase)	2.05 (1.30–3.47)	0.002
Use of icodextrin	2.74 (0.75–12.02)	0.130
Number of peritonitis (per 1-episode increase)	1.54 (0.98–2.41)	0.061
**B. Pathological predictors for EPS**		
	Univariable Logistic Regression	
Factors	OR (95%CI)	*P*
Thickness of peritoneal membrane (per 1-μm increase)	1.003 (1.00–1.01)	0.045
CD68-positive cells (per 1-cell increase)	0.99 (0.93–1.03)	0.514
New membrane formation (per 1-score increase)	2.18 (1.26–3.83)	0.006
D2-40 expression (per 1-score increase)	2.00 (0.87–4.39)	0.100
Presence of mesothelial cells	0.99 (0.59–1.64)	0.971
Perivascular bleeding	2.27 (0.38–10.15)	0.334
L/V ratio (per 0.1 increase)	0.43 (0.25–0.65)	<0.001
Presence of CD31-negative vessels	4.92 (1.28–19.22)	0.021
Fibrin deposition	28.60 (5.53–194.56)	<0.001
AGEs score (per 1-score increase)	2.86 (0.93–11.19)	0.068
Collagen volume fraction	1.05 (1.01–1.09)	0.018

OR, odds ratio; CI, confidence interval; PD, peritoneal dialysis; L/V ratio, ratio of luminal diameter to vessel diameter; AGEs, advanced glycation end-products; D2-40 is same as podoplanin.

### Pathological findings at PD catheter removal as predictors for EPS

Independent pathological predictive findings were investigated using the same methods (Table 2B). Univariable analysis revealed that the pathological findings in the peritoneal membrane for the predictive findings of EPS were: thickness of peritoneal membrane (OR 1.003, *P* = 0.045, Fig 2J), new membrane formation score (per 1-score increase, OR 2.18, *P* = 0.006, Fig 2B), L/V ratio (per 0.1 increase, OR 0.43, *P*<0.001, Fig 2D), presence of CD31-negative vessels (OR 4.92, *P* = 0.021, Fig 2E and 2F), fibrin deposition (OR 28.60, *P*<0.001, Fig 2H), and collagen volume fraction (OR 1.05, *P* = 0.018, Fig 3). Multivariable logistic regression analysis suggested that L/V ratio (per 0.1 increase, OR 0.50 [95%CI 0.29–0.78], *P* = 0.002) and fibrin deposition (OR 8.45 [95%CI 1.34–65.38], *P* = 0.023) could be the independent predictive findings for EPS (S2 Table).

### Analyses of samples with/without EPS matched for PD treatment period, non-diabetes, and PD solution (acidic or neutral pH)

**Fig 4:**
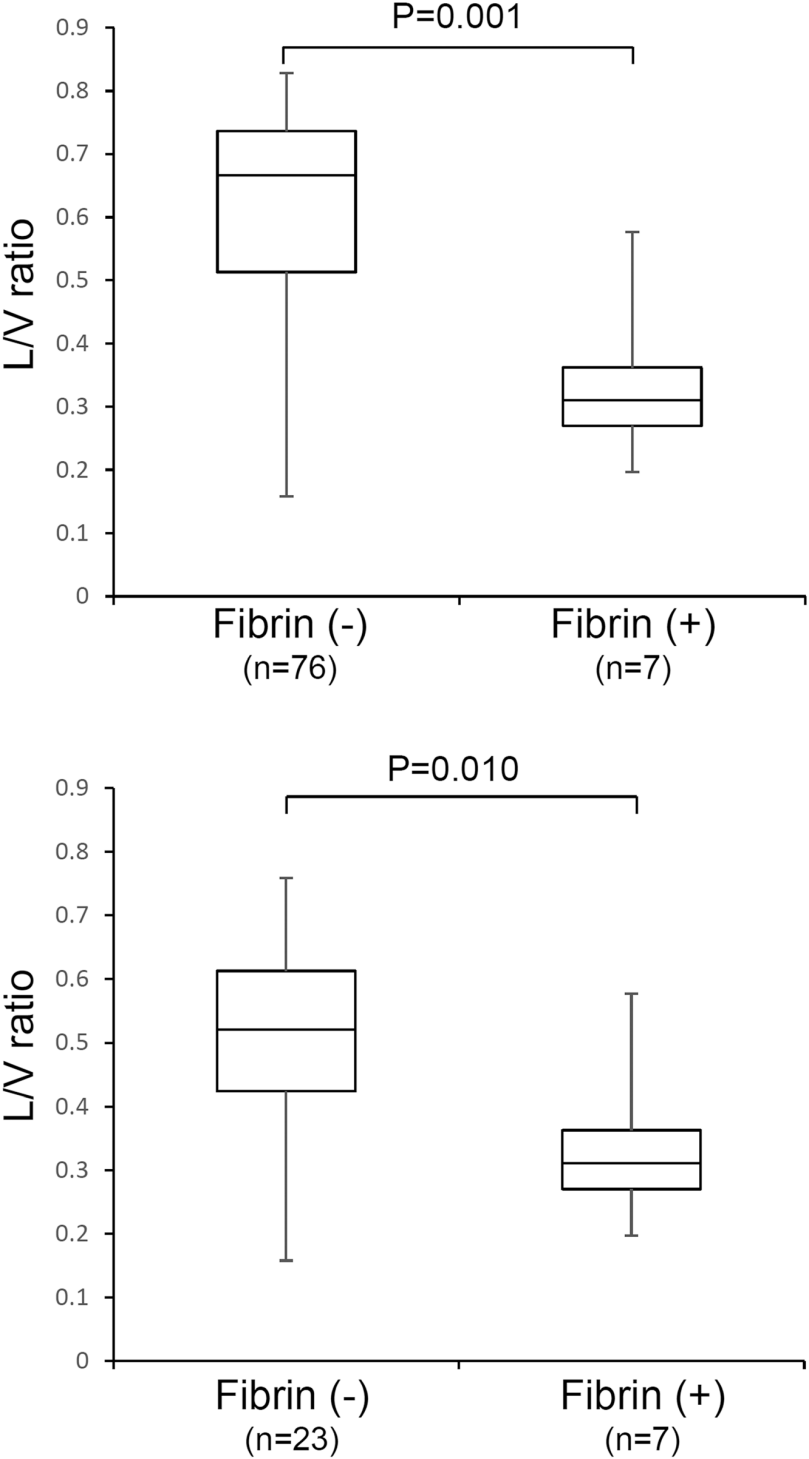
L/V ratio was lower in PD patients with fibrin exudation than in those without fibrin exudation. A, **cohort of** Table 1 **(n = 83),** B: **cohort of** Table 3 **(n = 30).**

Similar characteristics in the matched two groups were young age, long-term PD treatment (approximately 10-year treatment period), non-diabetic nephropathy, acidic solution, and low peritonitis occurrence rate (Table 3). The peritonitis rate was 0.1 episodes per patient per year in both groups. Univariable analysis (Table 4) identified the factors that were associated with EPS, including L/V ratio (per 0.1 increase, OR 0.44, *P* = 0.003) and fibrin deposition (OR 6.35, *P* = 0.027). Multivariable logistic regression analysis suggested that L/V ratio could be an independent pathological marker promoting EPS (per 0.1 increase, OR 0.18 [95%CI 0.00–0.70], *P* = 0.004) (S3 Table). Interestingly, L/V ratio was lower in patients with fibrin exudation than in those without fibrin exudation in both cohorts (Fig 4).

**Table 3:** Differences in general characteristics and pathological findings between the EPS and non-EPS groups matched for PD treatment period, non-diabetes and PD solution (acidic or neutral pH).

	non-EPS (n = 20)	EPS (n = 10)	P-value
Clinical factors			
Age (years)	46.4±9.58	41.2±14.34	0.251 †
Male, n (%)	11 (55.0)	8 (80.0)	0.246 *
Primary kidney disease			
Chronic glomerulonephritis, n (%)	11 (55.0)	8 (80.0)	0.246 *
Diabetes nephropathy, n (%)	0 (0.0)	0 (0.0)	
Acidic PD solution, n (%)	20 (100.0)	10 (100.0)	
PD duration (months)	127 (93.7–140.9)	128 (89.0–149.0)	0.792 ‡
Number of peritonitis (per patient)	0.5 (0.0–1.5)	1 (0.0–4.0)	0.283 ‡
Peritonitis rate (patient^-1^ × year^-1^)	0.0 (0.00–0.13)	0.1 (0.00–0.25)	0.313 ‡
Use of icodextrin, n (%)	9 (45.0)	7 (70.0)	0.260 *
Glucose exposure score at cessation of PD	2.0 (1.0–3.0)	3.0 (2.0–4.0)	0.106 ‡
Peritoneal lavage, n (%)	19 (95.0)	7 (70.0)	0.095 *
Steroid treatment, n (%)	4 (20.0)	3 (30.0)	0.657 *
Reason for withdrawal from PD			
Dialysis failure/UF failure, n (%)	4 (20.0)	7 (70.0)	0.015 *
Prevention of EPS, n (%)	16 (80.0)	3 (30.0)	
Pathological factors			
Thickness of peritoneal membrane (μm)	336.2 (262.79–455.75)	550.5 (227.77–750.00)	0.202 ‡
CD68-positive cells (number/mm)	13.6 (4.21–20.70)	8.6 (0.56–15.42)	0.312 ‡
Presence of mesothelial cells (grade)	1.0 (0.0–3.0)	1.0 (0.0–3.0)	0.591 ‡
New membrane formation (grade)	0.0 (0.0–2.0)	1.5 (0.0–2.5)	0.306 ‡
D2-40 expression (grade)	1.0 (0.0–2.0)	0.5 (0.0–1.5)	0.737 ‡
CD31 negative vessels, positive case (%)	7 (35.0)	5 (50.0)	0.461 *
Ratio of luminal diameter to vessel diameter (L/V ratio)	0.6 (0.43–0.61)	0.3 (0.26–0.43)	0.002 ‡
Fibrin deposition, positive case (%)	2 (10.0)	5 (50.0)	0.026 *
Perivascular bleeding, positive case (%)	2 (10.0)	2 (20.0)	0.584 *
AGEs score (grade)	2.0 (1.5–2.5)	2.8 (1.5–3.0)	0.203 ‡
Collagen volume fraction (%)	18.4 (11.50–24.20)	30.9 (25.50–48.00)	0.031 ‡

EPS, encapsulating peritoneal sclerosis; PD, peritoneal dialysis; AGEs, advanced glycation end-products; D2-40 is the same as podoplanin.

*n (%), Fisher's exact test;

^†^ mean±SD, Student’s t-test;

^‡^ median (IQR), Mann-Whitney's U-test

**Table 4:** Logistic regression analysis of clinical and pathological predictors for EPS in the cohort of Table 3.

**A. Clinical predictors for EPS**		
	Univariable Logistic Regression	
Factors	OR (95%CI)	*P*
Age (per 1-year increase)	0.97 (0.91–1.03)	0.284
Male	2.24 (0.56–12.73)	0.271
Chronic glomerulonephritis	2.47 (0.57–14.34)	0.233
Peritoneal lavage	0.09 (0.00–1.05)	0.056
Glucose exposure score at cessation of PD (per 1-score increase)	1.83 (0.89–5.80)	0.112
Use of icodextrin	2.47 (0.57–14.34)	0.234
Number of peritonitis (per 1-episode increase)	1.43 (0.85–2.88)	0.179
**B. Pathological predictors for EPS**		
	Univariable Logistic Regression	
Factors	OR (95%CI)	*P*
Thickness of peritoneal membrane (per 1-μm increase)	1.00 (1.00–1.01)	0.062
CD68-positive cells (per 1-cell increase)	0.98 (0.92–1.03)	0.464
New membrane formation (per 1-score increase)	1.51 (0.74–3.40)	0.260
D2-40 expression (per 1-score increase)	0.91 (0.35–2.43)	0.844
Presence of mesothelial cells	0.92 (0.54–1.52)	0.747
Perivascular bleeding	2.00 (0.31–12.94)	0.443
L/V ratio (per 0.1 increase)	0.44 (0.18–0.78)	0.003
Presence of CD31-negative vessels	1.69 (0.41–7.81)	0.472
Fibrin deposition	6.35 (1.22–62.62)	0.027
AGE score (per 1-score increase)	2.11 (0.53–10.41)	0.296
Collagen volume fraction	1.02 (0.99–1.05)	0.121

OR, odds ratio; CI, confidence interval; PD, peritoneal dialysis; L/V ratio, ratio of luminal diameter to vessel diameter; AGE, advanced glycation end-product; D2-40 is same as podoplanin.

## Discussion

The purpose of the present study was to identify the chronic peritoneal injuries found in peritoneal membrane biopsy tissues at PD catheter removal that promote EPS. Three factors are involved in the development of peritoneal damage, including fibrosis and vascular injury: 1) bioincompatible factors in PD fluid; 2) peritonitis, particularly repeated episodes of peritonitis; and 3) systemic inflammation related to uremia [37]. In this cohort with a low incidence of peritonitis, the main contributor to peritoneal injury was considered to be bioincompatibility of the peritoneal dialysis solution. Peritonitis with severe peritoneal injuries, particularly fungus and *Pseudomonas aeruginosa*-induced peritonitis, is known to be a high-risk factor for EPS [6,38–40]. Severe peritonitis is often associated with adhesions in the peritoneal cavity [36,41]. We confirmed that these organisms can induce fibrin exudation associated with strong inflammation (Fig 1), which can accelerate the development of bowel adhesions leading to EPS [42,43]. We found that vascular endothelial cell damage, such as that shown by low L/V ratio and fibrin exudation, is a pathological finding for the development of EPS. Nakayama proposed the plasma leak-to-response hypothesis that vascular alterations in the peritoneum of long-term PD patients play a crucial role in the initiation and development of EPS [44]. Fibrin exudation and new membrane formation are considered to be derived from high vascular permeability due to endothelial injury with severe vasculopathy [19]. Loss of vascular endothelial cell integrity, such as decrease of constitutive NOS-derived nitric oxide and glycocalyx, pericyte detachment, and vascular basal lamina degradation, have been reported to lead to plasma and fibrin exudation [45–48] which induce adhesion.

An early and disproportionate reduction in osmotic conductance due to submesothelial interstitial fibrosis was recently reported by Morelle et al. as predictive of EPS [29]. They suggested that impaired water transport due to strong interstitial fibrosis was predictive of EPS development. Our analyses found that collagen density tended to be higher in the EPS development group (Tables 1–4). Morelle et al. suggested that vasculopathy was not an important factor, but was prone to be severe in the EPS development group (n = 7) when compared with the control-matched long-term PD group (n = 7) [29], although the duration of PD treatment (62 months) was shorter than that in the present study (128 months).

The concept of vascular endothelial damage as an important factor triggering the development of EPS is in agreement with the many clinical factors of EPS reported previously. Patients with a high level of peritoneal membrane transporters are known to be at high risk for EPS [7–10]. In such patients, a glucose-based PD solution can be more easily absorbed through the capillaries and be exposed to the endothelial cells of capillaries and postcapillary venules than in patients having low levels of peritoneal transporters. Furthermore, patients with high levels of peritoneal transporters need many bags of high-concentration glucose-based PD solution in order to have sufficient net ultrafiltration volume to maintain fluid balance. Long-term PD treatment [2,4–6], a risk factor for EPS, exacerbates damage to the vascular endothelial cells by absorption of large amounts of PD solution. These clinical factors are thus often associated with the occurrence of EPS.

In contrast to glucose-based acidic PD solution, acidic icodextrin solution was not associated with the occurrence of EPS (Tables 1–4). Icodextrin has been reported to be absorbed mainly through lymphatic vessels from the peritoneal cavity because of the large size of the molecule [49]. In this regard, the impact of damage to vascular endothelial cells may be lower using an acidic icodextrin solution than an acidic glucose-based solution, which can be absorbed via the capillaries. In addition, vasculopathy has been reported to be less severe in patients treated with pH-neutral solution than in patients who undergo treatment with an acidic solution [32,50]. Based on these findings, we conclude that vascular endothelial injury due mainly to bioincompatibility of the PD solution might be an important trigger for the development of EPS.

Limitations must be considered when interpreting the present results. First, this was a retrospective cohort study. The number of patient samples was limited, which was also the situation in two recent studies with 7 and 9 patients who developed EPS [9,29], due to the rarity of this pathology. We performed multivariable logistic regression analysis and found that L/V ratio may offer a predictor for the development of EPS (S2 Table and Table 3). However, careful evaluation of these data is warranted, given the low frequency of EPS in this cohort. Second, all patients in the present study were Japanese, so genetic trends could not be evaluated. Genetic background may be related to susceptibility for the development of EPS [1]. Third, medications other than corticosteroids, such as renin angiotensin system inhibitors, were not evaluated. Fourth, frequency and volume of the peritoneal dialysis fluid for peritoneal lavage before PD catheter removal were variable, thus the precise methods were not evaluated. Fifth, CT was not performed at the cessation of PD in most patients. Sixth, we used glucose exposure at the cessation of PD because assessment of total glucose exposure was not feasible. Seventh, D/P Cr performed within 6 months before catheter removal tended to be higher in the EPS development group than in the non-EPS development group (0.79±0.13 in EPS development group, 0.70±0.17 in non-EPS development group, *P* = 0.285), but PET was only performed in 52% of patients within 6 months before catheter removal. Eighth, this cohort was characterized by a low frequency of peritonitis. In this respect, the findings for this cohort may not be generalizable to the wider population of PD patients.

In summary, L/V ratio and fibrin deposition in the peritoneal membrane sampled at the time of PD catheter removal are associated with the development of EPS, especially in long-term PD patients with a low frequency of peritonitis. Vascular endothelial damage in the peritoneal membrane, which can induce vascular leakage of fibrin leading to EPS, could be a predictive finding of EPS in long-term PD patients. Future prospective studies in a large cohort are necessary to verify these results.

## Supporting Information

S1 Fig Flow diagram of the study population. (PDF)

S2 Fig Definition of the pathological findings. (PDF)

S3 Fig Representative pathological findings and definitions. (PDF)

S4 Fig Proposed mechanisms of development of EPS and possible predictors of EPS. (PDF)

S1 Table List of antibodies used. (PDF)

S2 Table Logistic regression analysis of clinical and pathological predictors for EPS. (PDF)

S3 Table Conditional logistic regression with Firth’s bias correction of clinical and pathological predictors for EPS. (PDF)
