# Supplementary material for: Vascular Endothelial Cell Injury Is an Important Factor in the Development of Encapsulating Peritoneal Sclerosis in Long-Term Peritoneal Dialysis Patients
Source: PLoS One. 2016 Apr 27;11(4):e0154644. doi: 10.1371/journal.pone.0154644 (PMC4847858; doi:10.1371/journal.pone.0154644)
Supplement: S1 Fig — (PDF) [file pone.0154644.s001.pdf]

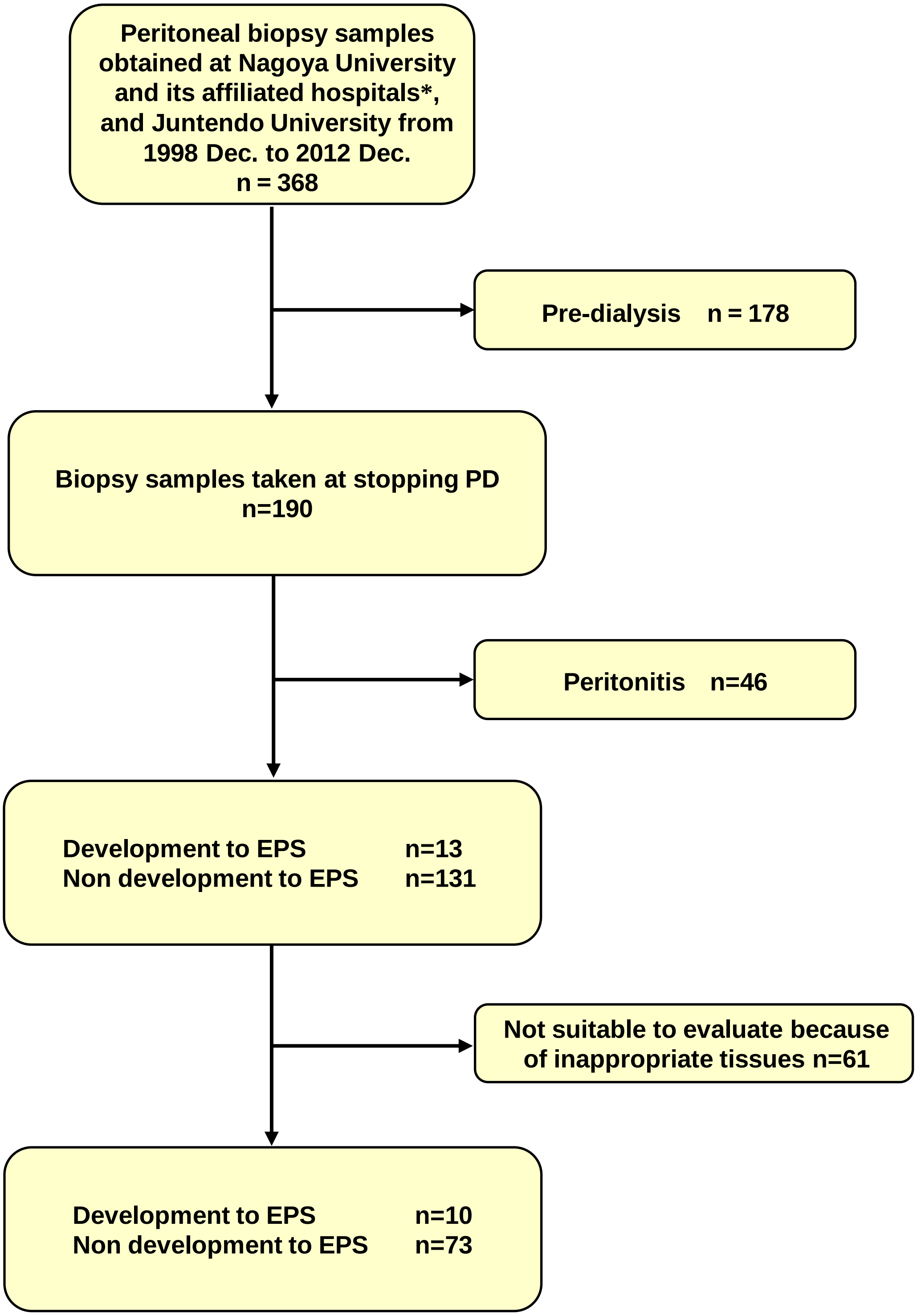

S1 Fig. Flow diagram of the study population.

* Affiliated hospitals include Handa Municipal Hospital (Handa, Japan), Yokkaichi Municipal Hospital (Yokkaichi, Japan), Nagoya Kyoritsu Hospital (Nagoya, Japan), Chubu Rosai Hospital (Nagoya, Japan), Toyota-Kousei Hospital (Toyota, Japan), Daiyukai-Daiichi Hospital (Ichinomiya, Japan), Anjyo-Kousei Hospital (Anjyo, Japan), Kasugai Municipal Hospital (Kasugai, Japan)

Supplementary Figure 1
