# Supplementary material for: Vascular Endothelial Cell Injury Is an Important Factor in the Development of Encapsulating Peritoneal Sclerosis in Long-Term Peritoneal Dialysis Patients
Source: PLoS One. 2016 Apr 27;11(4):e0154644. doi: 10.1371/journal.pone.0154644 (PMC4847858; doi:10.1371/journal.pone.0154644)
Supplement: S2 Fig — (PDF) [file pone.0154644.s002.pdf]

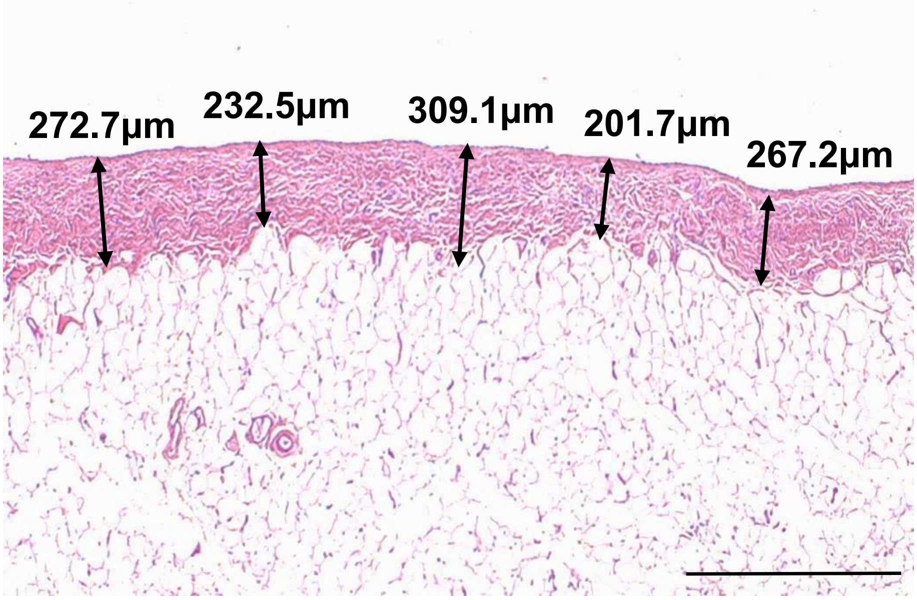

A. Measurement of the peritoneal membrane thickness

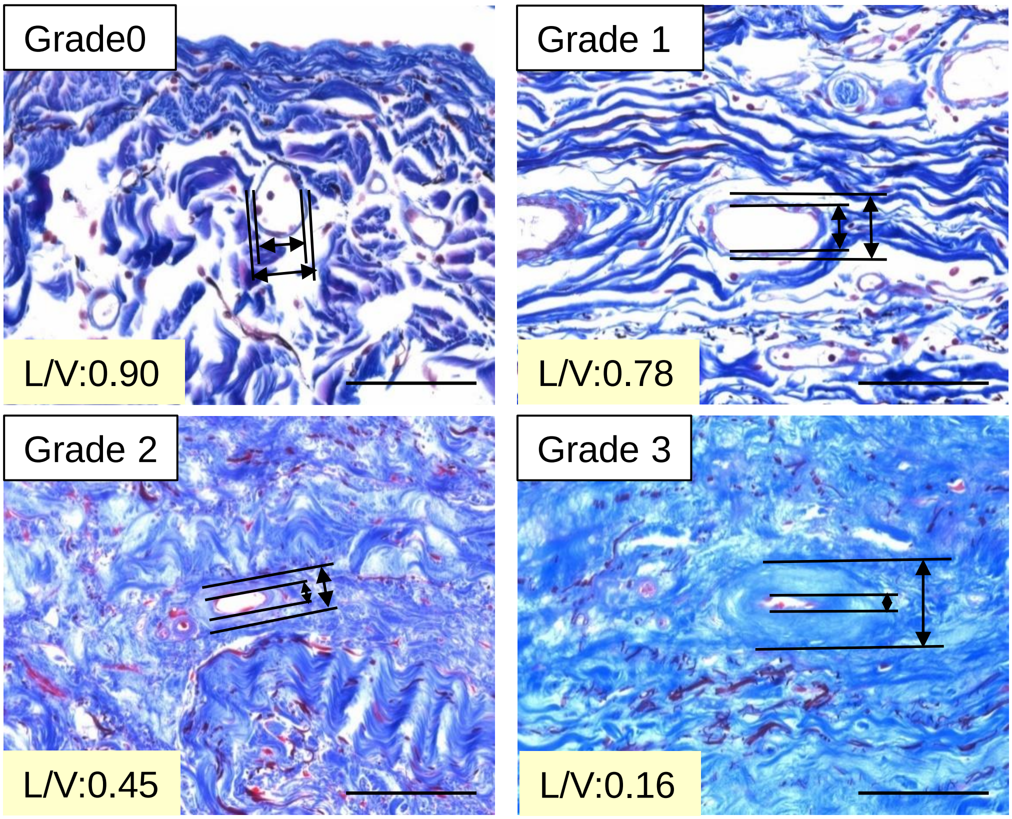

B. Assessment of the vasculopathy

S2 Fig. Definition of the pathological findings (1)

(A) Peritoneal thickening: In order to assess the extent of peritoneal thickening, the submesothelial compact zone was defined and thickness was measured at 5 points. Then, their average was calculated. Scale bar = 500 μm. **(B) Vasculopathy:** Vasculopathy was assessed by the ratio of luminal diameter (L) to vessel diameter (V), which was defined as diameter of lumen/diameter of vessel ratio (L/V ratio). Scale bars = 100 μm.

Supplementary Figure 2-1

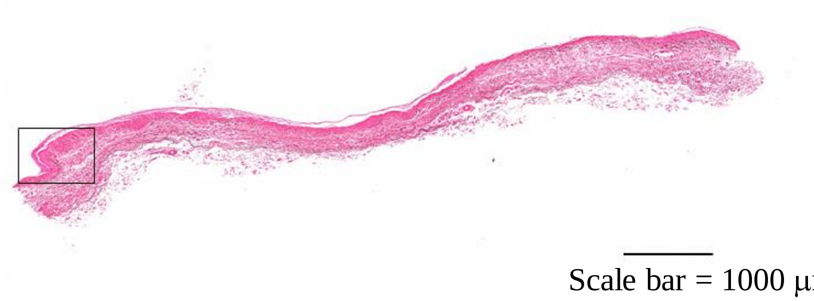

Scale bar = 1000 μm

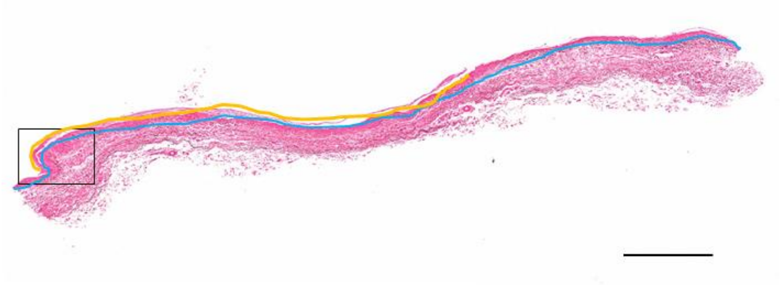

Scale bar = 1000 μm

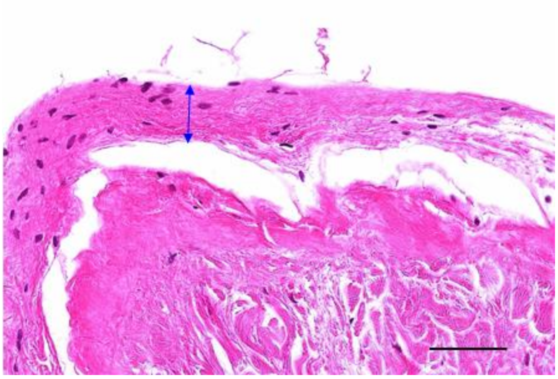

Scale bar = 50 μm

Positive % of the surface length
= length of blue line / length of yellow line
=804μm / 5951μm
=61 % (grade 3)

Average thickness of new membrane
= 39 μm (grade 1)

"new membrane formation score"
= (3 + 1)/2= 2

C. New membrane formation score

S2 Fig. Definition of the pathological findings (2)

(C) New membrane formation score: New membrane formation was assessed by positive percentage of surface length and thickness. Positive percentage of surface length was graded into 4 groups: (0) 0%; (1) >0% and ≤25%; (2) >25% and ≤50%; and (3) >50% and ≤100%. Thickness of the new membrane was graded into 4 groups: (0) 0; (1) >0 and ≤100 μm; (2) >100 and ≤250 μm; and (3) >250 μm.
The average of the grades was the new membrane formation score.

Supplementary Figure 2-2

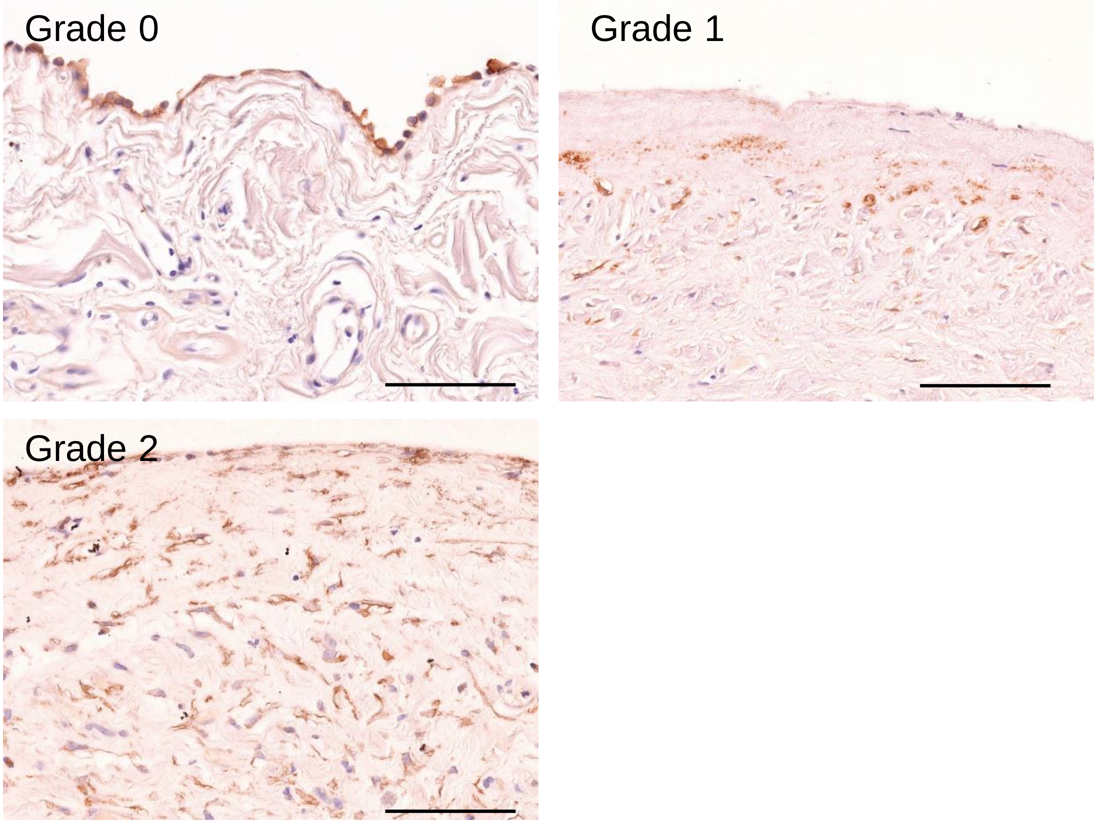

D. Assessment of the podoplanin (D2-40) expression

S2 Fig. Definition of the pathological findings (3)

(D) Podoplanin (D2-40) expression in peritoneal membranes:

Podoplanin (D2-40)-positive cells were semi-quantitatively classified into three groups according to the reports by Braun [18]: 0) positive podoplanin staining on lymphatics and mesothelial cells, but not on single cells with fibroblastic appearance; 1) focal accumulation of podoplanin-positive cells with fibroblastic appearance; and 2) diffuse accumulation of podoplanin-positive cells with fibroblastic appearance. Scale bars = 100 μm.

Supplementary Figure 2-3

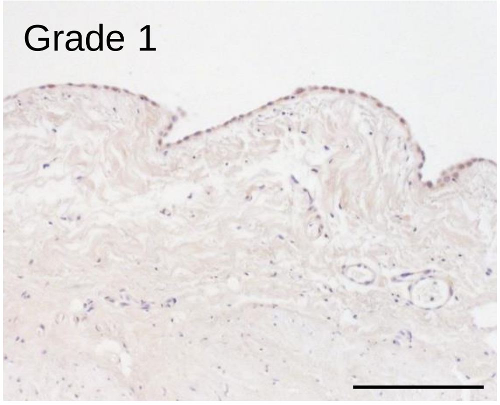

Interstitial AGEs accumulation (1)
Vascular AGEs accumulation (1)

AGEs accumulation score
= (1+ 1)/2= Grade 1

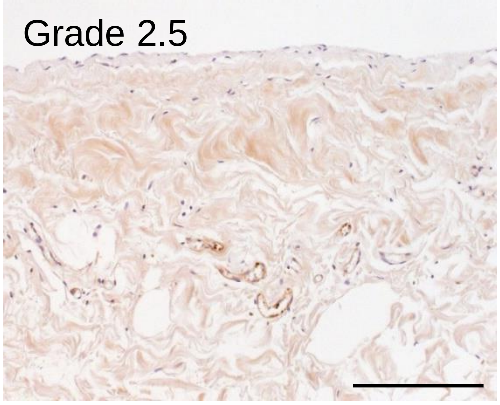

Interstitial AGEs (2)
Vascular AGEs (3)

AGEs accumulation score
= (2+ 3)/2= Grade 2.5

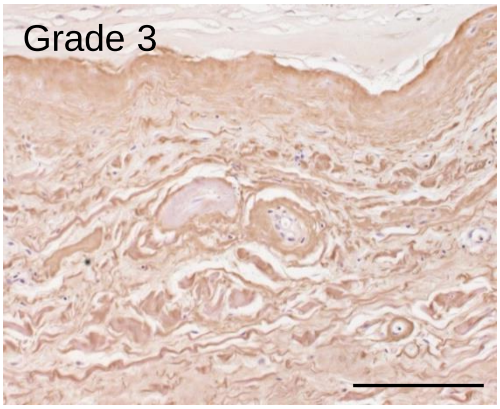

Interstitial AGEs (3)
Vascular AGEs (3)

AGEs accumulation score
= (3+ 3)/2= Grade 3

E. Assessment of the AGEs accumulation score

S2 Fig. Definition of the pathological findings (4)

(E) Advanced glycation end-products (AGEs) accumulation score: AGEs accumulation was analyzed in the interstitial area and in the vessels walls separately, and was semi-quantitatively classified into four groups based on the intensity of the positive staining: (0) no staining; (1) mild staining; (2) moderate staining; and (3) pronounced staining. The average of the scores was calculated and defined as the AGEs accumulation score. Three examples to calculate the scores are shown. Scale bars = 200μm.

Supplementary Figure 2-4
