# Supplementary material for: Vascular Endothelial Cell Injury Is an Important Factor in the Development of Encapsulating Peritoneal Sclerosis in Long-Term Peritoneal Dialysis Patients
Source: PLoS One. 2016 Apr 27;11(4):e0154644. doi: 10.1371/journal.pone.0154644 (PMC4847858; doi:10.1371/journal.pone.0154644)
Supplement: S3 Fig — (PDF) [file pone.0154644.s003.pdf]

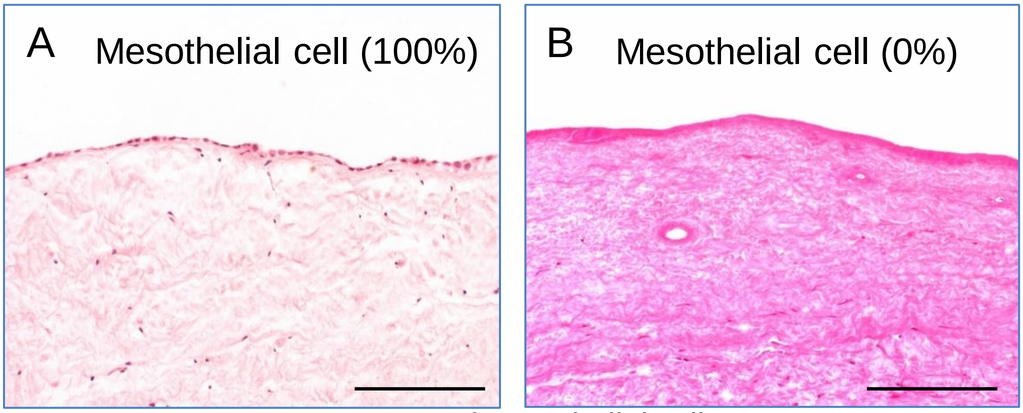

Presence of mesothelial cells

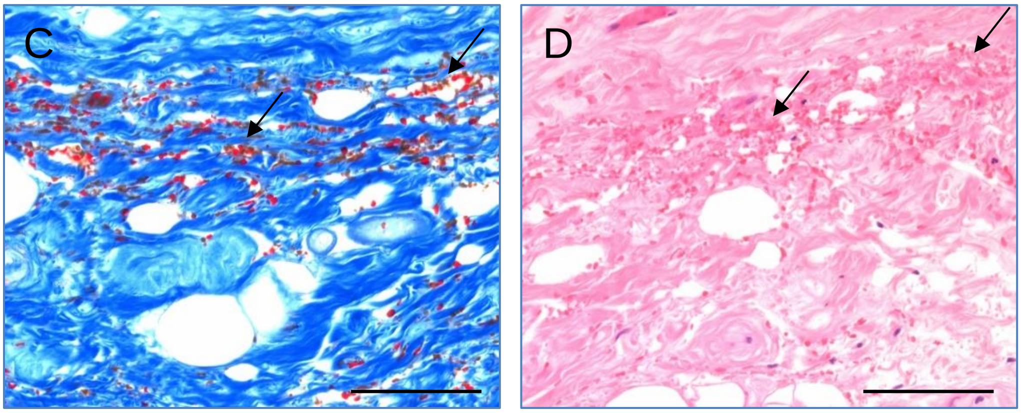

Positivity of perivascular bleeding

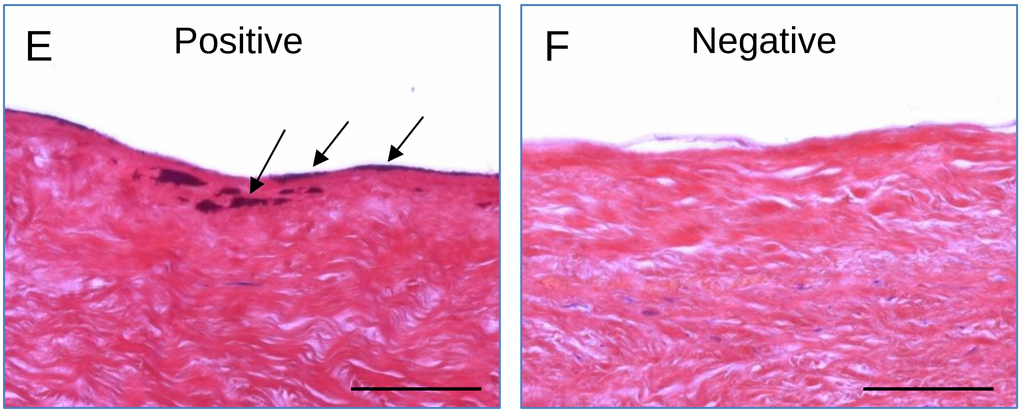

Assessment of presence of fibrin deposition

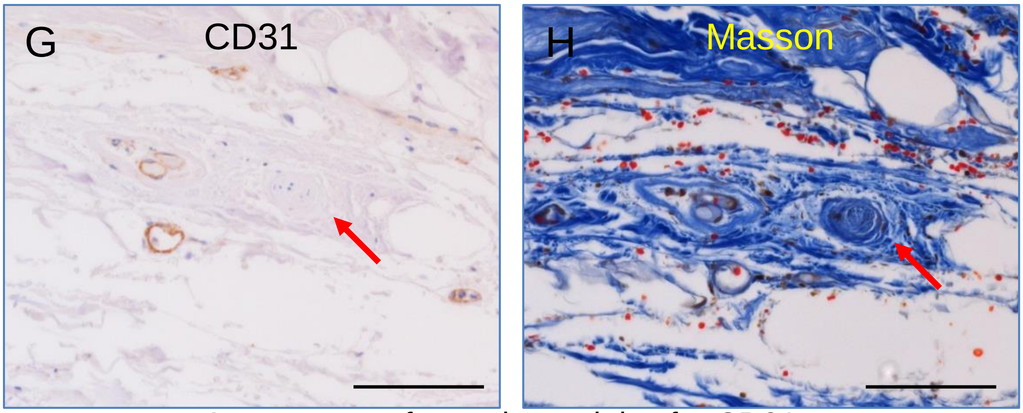

Assessment of negative staining for CD31

Supplementary Figure 3-1

Supplementary Figure 3. Representative pathological findings and definitions.
A, B: The proportion of mesothelial cells was assessed by positive percentage of surface length, and was graded into 4 groups: (0) 0%; (1) >0% and ≤25%; (2) >25% and ≤50%; and (3) >50% and ≤100%.
C, D: Perivascular bleeding. Arrows indicate perivascular bleeding.
E, F: Positivity of fibrin deposition was detected by PTAH staining. Arrows indicate fibrin exudation.
G, H: Negative staining of CD31 in blood vessels was assessed as presence (+) or absence (–). Arrow indicates negative expression for CD31 in the vessels.
A, **B**, **D**, Hematoxylin and eosin (HE) staining; **C**, **H**, Masson's trichrome stain; **E**, **F**, PTAH staining; **G**, CD31 immunostaining
Scale bars in A and B = 200 µm. Scale bars in C to H = 100 µm.
PTAH, phosphotungstic acid hematoxylin

Supplementary Figure 3-2
