# Supplementary figures and images for: Vascular Endothelial Cell Injury Is an Important Factor in the Development of Encapsulating Peritoneal Sclerosis in Long-Term Peritoneal Dialysis Patients
Source: PLoS One. 2016 Apr 27;11(4):e0154644. doi: 10.1371/journal.pone.0154644 (PMC4847858; doi:10.1371/journal.pone.0154644)

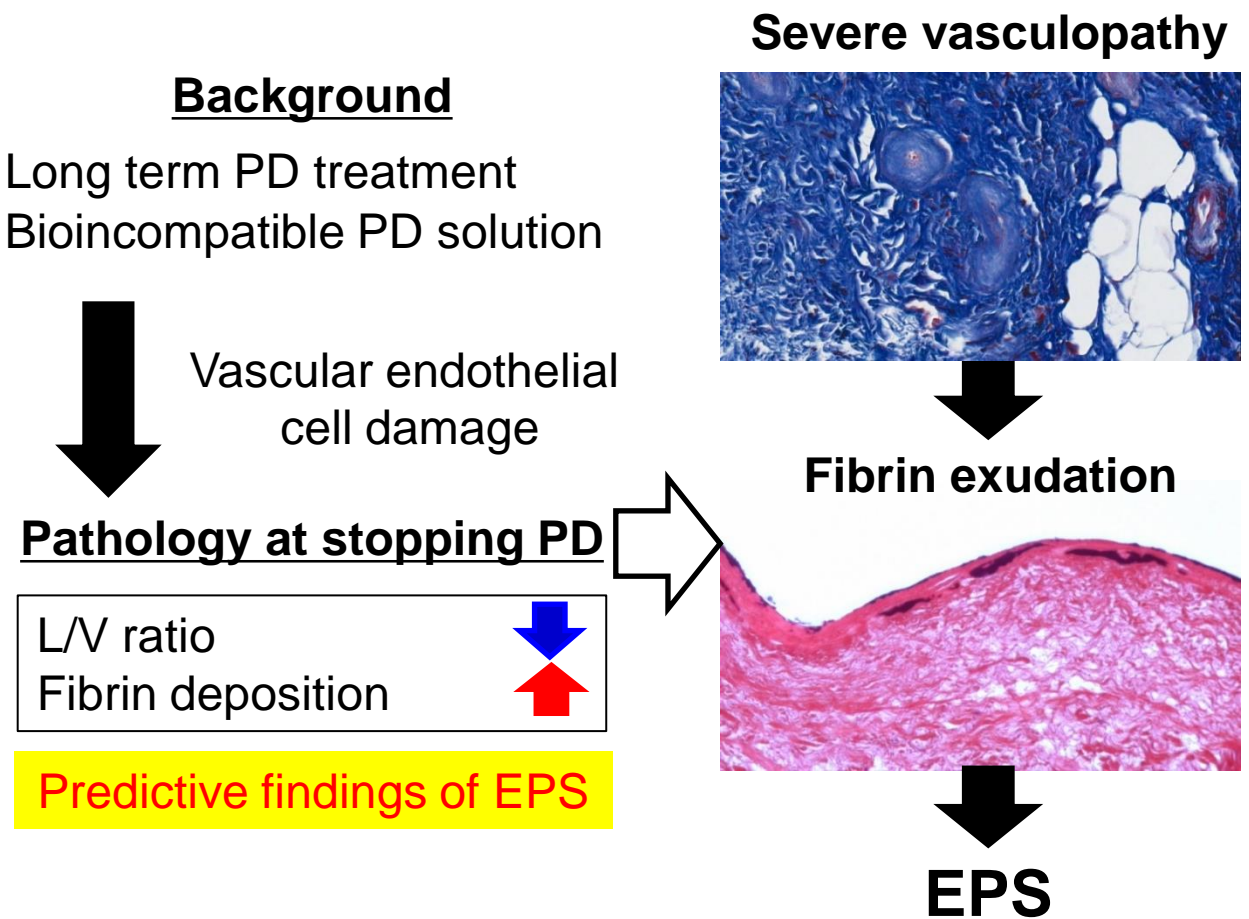

**S4 Fig.** Proposed mechanisms of development of EPS and possible predictors of EPS.

Supplement: S4 Fig — (PDF) [file pone.0154644.s004.pdf]
