# Supplementary material for: Vascular Endothelial Cell Injury Is an Important Factor in the Development of Encapsulating Peritoneal Sclerosis in Long-Term Peritoneal Dialysis Patients
Source: PLoS One. 2016 Apr 27;11(4):e0154644. doi: 10.1371/journal.pone.0154644 (PMC4847858; doi:10.1371/journal.pone.0154644)
Supplement: S1 Table — (PDF) [file pone.0154644.s005.pdf]

List of antibodies used

Antibody	Company
mouse anti-human CD31 antibody	Dako, Glostrup, Denmark
mouse anti-human CD68 antibody	Dako, Glostrup, Denmark
monoclonal mouse anti-human D2-40 antibody	Dako, Glostrup, Denmark
anti-AGEs monoclonal antibody	Trans Genic, Kobe, Japan
goat anti-mouse IgG antibody	Nichirei, Tokyo, Japan
DAB (3,3-diaminobenzidine)	Nichirei, Tokyo, Japan

Supplementary Table 1
