# Supplementary material for: Vascular Endothelial Cell Injury Is an Important Factor in the Development of Encapsulating Peritoneal Sclerosis in Long-Term Peritoneal Dialysis Patients
Source: PLoS One. 2016 Apr 27;11(4):e0154644. doi: 10.1371/journal.pone.0154644 (PMC4847858; doi:10.1371/journal.pone.0154644)
Supplement: S2 Table — (PDF) [file pone.0154644.s006.pdf]

• Clinical predictors for EPS

	Univariable Logistic Regression		Multivariable Logistic Regression	
Factors	OR (95%CI)	*P*	OR (95%CI)	*P*
Age (per 1-year increase)	0.93 (0.87-0.98)	0.007	0.95 (0.88-1.01)	0.081
PD duration (per 1-month increase)	1.02 (1.01-1.04)	0.003		
Diabetes nephropathy	0.11 (0.00-0.91)	0.039	0.10 (0.00-1.22)	0.077
Acidic PD solution	15.57 (1.88-2029.29)	0.006		
Peritoneal lavage	1.68 (0.46-7.37)	0.443		
Steroid treatment	5.81 (1.16-27.14)	0.034	4.16 (0.63-30.08)	0.136
Glucose exposure score at cessation of PD (per 1-score increase)	2.05 (1.30-3.47)	0.002	2.03 (1.17-3.96)	0.011
Use of icodextrin	2.74 (0.75-12.02)	0.130		
Number of peritonitis (per 1-episode increase)	1.42 (0.81-2.45)	0.213		

• Pathological predictors for EPS

	Univariable Logistic Regression		Multivariable Logistic Regression	
Factors	OR (95%CI)	*P*	OR (95%CI)	*P*
Thickness of peritoneal membrane (per 1-μm increase)	1.003 (1.00-1.01)	0.045		
CD68-positive cells (per 1-cell increase)	0.99 (0.93-1.03)	0.514		
New membrane formation (per 1-score increase)	2.18 (1.26-3.83)	0.006		
D2-40 expression (per 1-score increase)	2.00 (0.87-4.39)	0.100		
Presence of mesothelial cells	0.99 (0.59-1.64)	0.971		
Perivascular bleeding	2.27 (0.38-10.15)	0.334		
L/V ratio (per 0.1 increase)	0.43 (0.25-0.65)	<0.001	0.50 (0.29-0.78)	0.002
Presence of CD31-negative vessels	4.92 (1.28-19.22)	0.021		
Fibrin deposition	28.60 (5.53-194.56)	<0.001	8.50 (1.34-65.38)	0.023
AGEs score (per 1-score increase)	2.96 (0.93-11.19)	0.068		
Collagen volume fraction	1.05 (1.01-1.09)	0.018		

OR, odds ratio; CI, confidence interval; PD, peritoneal dialysis; L/V ratio, ratio of luminal diameter to vessel diameter; AGEs, advanced glycation end-products; D2-40 is same as podoplanin.

S2 Table. Logistic regression analysis of clinical and pathological predictors for EPS.

Supplementary Table 2
