# Supplementary material for: Vascular Endothelial Cell Injury Is an Important Factor in the Development of Encapsulating Peritoneal Sclerosis in Long-Term Peritoneal Dialysis Patients
Source: PLoS One. 2016 Apr 27;11(4):e0154644. doi: 10.1371/journal.pone.0154644 (PMC4847858; doi:10.1371/journal.pone.0154644)
Supplement: S3 Table — (PDF) [file pone.0154644.s007.pdf]

• Clinical predictors for EPS

	Univariable Logistic Regression		Multivariable Logistic Regression	
Factors	OR (95%CI)	*P*	OR (95%CI)	*P*
Age (per 1-year increase)	0.97 (0.91-1.03)	0.284		
Male	2.24 (0.56-12.73)	0.271		
Chronic glomerulonephritis	2.47 (0.57-14.34)	0.233	4.19 (0.42-600.04)	0.232
Peritoneal lavage	0.09 (0.00-1.05)	0.056	0.11 (0.00-1.35)	0.086
Glucose exposure score at cessation of PD (per 1-score increase)	1.83 (0.89-5.80)	0.112	2.02 (0.89-8.73)	0.104
Use of icodextrin	2.47 (0.57-14.34)	0.234	2.73 (0.43-36.29)	0.304
Number of peritonitis (per 1-episode increase)	1.43 (0.85-2.88)	0.179		

• Pathological predictors for EPS

	Univariable Logistic Regression		Multivariable Logistic Regression	
Factors	OR (95%CI)	*P*	OR (95%CI)	*P*
Thickness of peritoneal membrane (per 1-μm increase)	1.00 (1.00-1.01)	0.062		
CD68-positive cells (per 1-cell increase)	0.98 (0.92-1.03)	0.464		
New membrane formation (per 1-score increase)	1.51 (0.74-3.40)	0.260		
D2-40 expression (per 1-score increase)	0.91 (0.35-2.43)	0.844		
Presence of mesothelial cells	0.92 (0.54-1.52)	0.747		
Perivascular bleeding	2.00 (0.31-12.94)	0.443		
L/V ratio (per 0.1 increase)	0.44 (0.18-0.78)	0.003	0.18(0.00-0.70)	0.004
Presence of CD31-negative vessels	1.69 (0.41-7.81)	0.472	0.05(0.00-1.63)	0.102
Fibrin deposition	6.35 (1.22-62.62)	0.027		
AGE score (per 1-score increase)	2.11 (0.53-10.41)	0.296		
Collagen volume fraction	1.02 (0.99-1.05)	0.121		

OR, odds ratio; CI, confidence interval; PD, peritoneal dialysis; L/V ratio, ratio of luminal diameter to vessel diameter; AGE, advanced glycation end-product; D2-40 is same as podoplanin.

S3 Table. Conditional logistic regression with Firth's bias correction of clinical and pathological predictors for EPS in the cohort of Table 3.

Supplementary Table 3
